# Non-Structural Carbohydrate Storage Strategy Explains the Spatial Distribution of Treeline Species

**DOI:** 10.3390/plants9030384

**Published:** 2020-03-20

**Authors:** Hudong Han, Hongshi He, Zhengfang Wu, Yu Cong, Shengwei Zong, Jianan He, Yuanyuan Fu, Kai Liu, Hang Sun, Yan Li, Changbao Yu, Jindan Xu

**Affiliations:** 1 Key Laboratory of Geographical Processes and Ecological Security in Changbai Mountains, Ministry of Education, School of Geographical Sciences, Northeast Normal University, Changchun 130024, China; hanhd254@nenu.edu.cn (H.H.); congy345@nenu.edu.cn (Y.C.); zongsw049@nenu.edu.cn (S.Z.);; 2 School of Natural Resources, University of Missouri, Columbia, MO 65211, USA; 3 Northeast Institute of Geography and Agricultural Ecology, Chinese Academy of Sciences, Changchun 130102, China; 4 Changbai Mountain Nature Conservation Management Center, Erdaobaihe 133613, China

**Keywords:** non-structural carbohydrates, alpine treeline, spatial distribution of species, Changbai Mountain

## Abstract

Environmental factors that drive carbon storage are often used as an explanation for alpine treeline formation. However, different tree species respond differently to environmental changes, which challenges our understanding of treeline formation and shifts. Therefore, we selected *Picea jezoensis* and *Betula ermanii*, the two treeline species naturally occurring in Changbai Mountain in China, and measured the concentration of non-structural carbohydrates (NSC), soluble sugars and starch in one-year-old leaves, shoots, stems and fine roots at different elevations. We found that compared with *P. jezoensis*, the NSC and soluble sugars concentrations of leaves and shoots of *B. ermanii* were higher than those of *P. jezoensis*, while the starch concentration of all the tissues were lower. Moreover, the concentration of NSC, soluble sugars and starch in the leaves of *B. ermanii* decreased with elevation. In addition, the starch concentration of *B. ermanii* shoots, stems and fine roots remained at a high level regardless of whether the soluble sugars concentration decreased. Whereas the concentrations of soluble sugars and starch in one-year-old leaves, shoots and stems of *P. jezoensis* responded similarly changes with elevation. These findings demonstrate that compared with *P. jezoensis*, *B. ermanii* has a higher soluble sugars/starch ratio, and its shoots, stems and fine roots actively store NSC to adapt to the harsh environment, which is one of the reasons that *B. ermanii* can be distributed at higher altitudes.

## 1. Introduction

The alpine treeline is the upper limit of mountain forest distribution and is highly sensitive to climate change and human disturbance [1,2]. With global warming, upward shifts of the alpine treeline have been observed in most areas [3,4,5,6,7], while some more drought-prone areas have remained unchanged, or even moved downward [8,9,10,11]. In most studies, all of these phenomena seem to be related to environmental factors [5,8,9,10,12]. However, different tree species vary in their responses to environmental changes [13]. As a result of the asynchrony of alpine treeline shift and climate change, an enormous challenge has been brought to predict future alpine treeline shifts. Therefore, studying two different treeline species in response to environmental changes will help to explain the formation and shifts of the alpine treeline.

Elevation usually causes drastic changes in environmental factors, such as temperature, water and light, which greatly affect trees’ growth and treeline formation [2,5]. This leads to adaptive changes in the species and physiological characteristics of the trees. Many studies have shown that low temperature is the main limiting factor for alpine treeline formation and can explain nearly 80% of the global variability of alpine treeline elevation [3,4,14]. Non-structural carbohydrates (NSC), including soluble sugars and starch, are critical energy involved in plant growth and metabolism [15,16,17]. In addition, NSC is also an important index of the physiological hypothesis, which indicates the formation of alpine treelines [18].

The NSC concentrations of tree tissues can reflect the balance between carbon sources (photosynthesis) and the carbon sink (respiration and tree growth) as well as mirror tree growth in response to environmental changes [19]. Different tree species have varying patterns of NSC storage and utilization strategies [20], which is an important reason why different species have different environmental tolerance and occupy different spatial areas along with elevational gradients. Higher NSC concentrations can improve the ability of trees to resist drought [21] and cold stress, which is conducive to the growth and survival of the trees in harsh environments [22]. When in severe environmental conditions, NSC storage can even prioritize over growth [22,23]. For example, Galiano et al. (2017) showed that during the drought recovery period, *Pinus sylvestris* had a “drought memory effect,” using more carbon for storage, whereas *Tilia platyphyllos* primarily used more carbon for growth [24]. In addition, the NSC concentration in deciduous trees is generally higher than that of evergreens [25]. As a result of insufficient photosynthesis due to frost and wind, deciduous tree species can use more NSC to maintain physiological metabolism and resist environmental stress than evergreen species [20,26].

Currently, most studies on alpine treeline formation and shifts primarily focus on the restriction of environmental factors in tree physiology [2,12], whereas there are fewer studies on the differences in NSC storage strategies between evergreen and broad-leaved tree species near treelines. On the western slope of Changbai Mountain, the upper limit of the deciduous broad-leaved *Betula ermanii* is 2200 m a.s.l. [27], while the highest distribution of the evergreen conifer *Picea jezoensis* is 1800 m a.s.l. With climate warming, the upper limit of their distribution may shift up to varying degrees [3]. Thus, understanding the physiological adaptation of different treeline species to environmental changes enables more effective predictions of the alpine treeline and tree species distribution shifts in the future.

In this study, we selected *P. jezoensis* and *B. ermanii*, the treeline species naturally occurred in Changbai Mountain, and measured the concentration of NSC, soluble sugars and starch in one-year-old leaves, shoots, stems and fine roots along with elevation. Our objectives were to determine (1) the differences in the concentrations of NSC, soluble sugars, starch and NSC storage strategies in different tissues of *B. ermanii* and *P. jezoensis*; and (2) the influence of these differences on treeline formation and the spatial distribution of tree species.

## 2. Results

### 2.1. Characteristics of NSC Concentrations in B. ermanii and P. jezoensis

**Figure 1:**
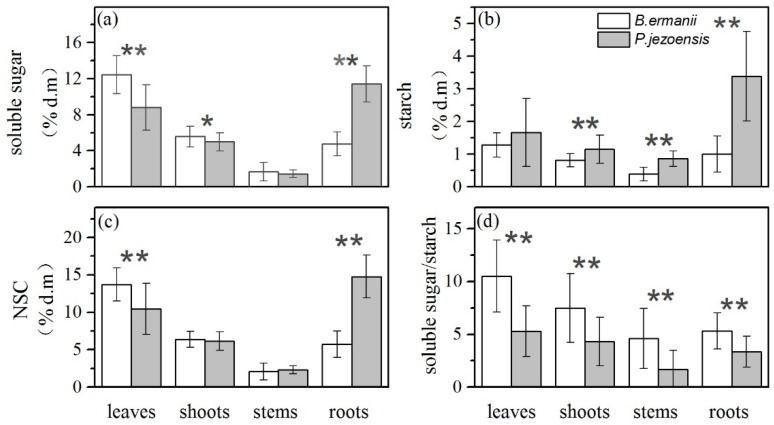
Concentrations of (**a**) soluble sugars, (**b**) starch, (**c**) NSC and (**d**) soluble sugar/starch ratio (mean ± SD) of all elevations for *B. ermanii* and *P. jezoensis* tissues. Asterisks (*, *p* < 0.05; **, *p* < 0.01) indicate statistically significant differences between *B. ermanii* and *P. jezoensis* with each tissue as determined by T-test.

**Figure 2:**
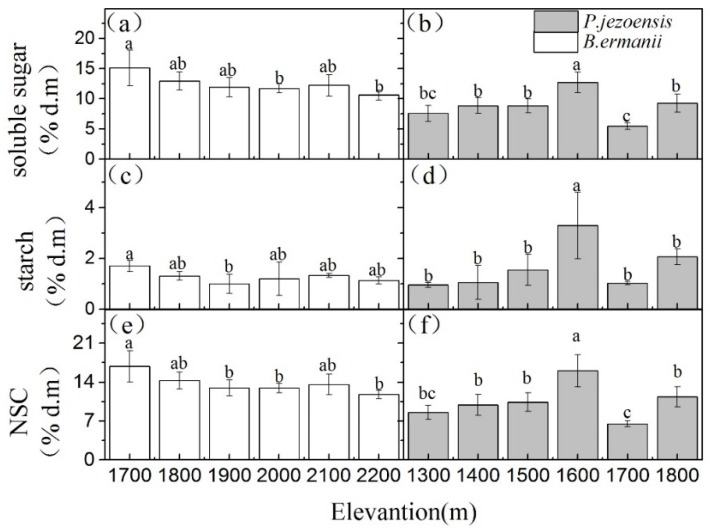
Variation in the concentrations of *B. ermanii* leaves (**a**) soluble sugar, (**c**) starch and (**e**) NSC as well as *P. jezoensis* leaves (**b**) soluble sugar, (**d**) starch and (**f**) NSC (mean ± SD; % of dry matter) along with elevational gradients in Changbai Mountain (*n* = 5 for each elevational site). Different letters indicate significant differences (*p* < 0.05) among elevations as determined by Tukey’s Honestly Significant Difference (HSD) test single-factor ANOVAs.

The concentrations of soluble sugars, starch, NSC and the soluble sugars/starch ratio were all significantly different in tree species, tissue types and their interactions (Table 1; *p* < 0.05). Compared with *P. jezoensis*, soluble sugars concentration of leaves and shoots in *B. ermanii* were higher, but soluble sugars concentrations in fine roots were lower (Figure 1a; *p* < 0.01). In addition, the starch concentration of *P. jezoensis* tissues was 1.5 times higher than that of *B. ermanii* (Figure 1b). Therefore, Leaves NSC concentration in *B. ermanii* was significantly higher than that in *P. jezoensis*, but fine roots NSC concentrations in *B. ermanii* was significlantly lower than that of *P. jezoensis* (Figure 2c; *p* < 0.01). Moreover, the soluble sugars/starch ratio in *B. ermanii* tissues was significantly higher than that of *P. jezoensis* (Figure 1d; *p* < 0.01).

### 2.2. Characteristics of NSC Concentrations in B. ermanii and P. jezoensis with Elevation Gradient

**Figure 3:**
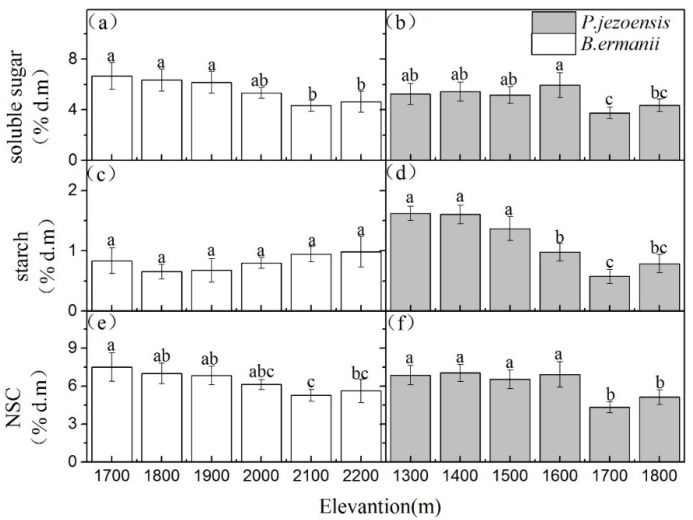
Variation in the concentrations of *B. ermanii* shoots (**a**) soluble sugar, (**c**) starch and (**e**) NSC as well as *P. jezoensis* shoots (**b**) soluble sugar, (**d**) starch and (**f**) NSC (mean ± SD; % of dry matter) along with elevational gradients in Changbai Mountain (*n* = 5 for each elevational site). Different letters indicate significant differences (*p* < 0.05) among elevations as determined by Tukey’s HSD test single-factor ANOVAs.

Different tissues NSC, soluble sugars and starch concentrations in *B. ermanii* and *P. jezoensis* showed different trends with elevation. In *B. ermanii*, except for a higher value in 1700m a.s.l., leaves NSC, soluble sugars and starch concentrations had a relatively stable across the elevational gradients. (Figure 2a,c,e; *p* < 0.05). As for *P. jezoensis*, one-year-old needles NSC, soluble sugars and starch concentrations were significantly higher at the medium elevantion (1600 m a.s.l.) than at the low elevation (1300 m and 1 400 m a.s.l.) and the high elevation (1700 m and 1800 m) (Figure 2b,d,f; *p* < 0.05). Shoots starch concentrations in *B. ermanii* were not significantly different at the various elevations (Figure 3c), whereas the concentrations of NSC and soluble sugars were higher at the lower elevation (1700 m and 1800 m a.s.l.) than at the higher elevation (2100 m and 2200 m a.s.l.) (Figure 3a,e). A similar pattern was observed in shoots NSC, soluble sugars and starch concentration of *P. jezoensis*, which tended to decrease with increasing elevation (Figure 3b,d,f; *p* < 0.05).

**Figure 4:**
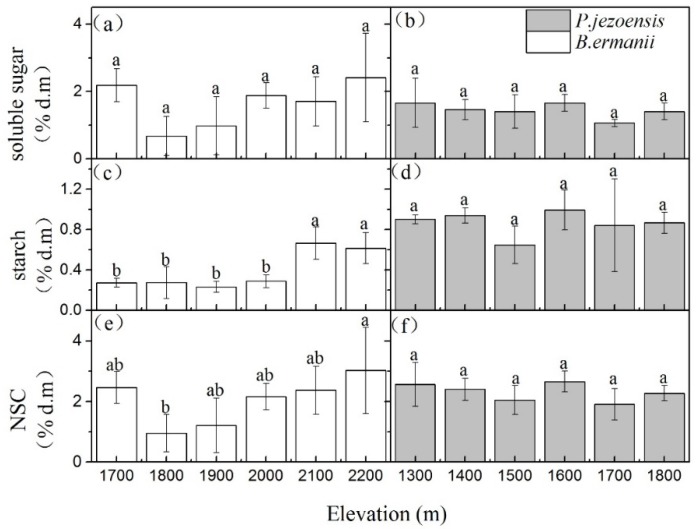
Variation in the concentrations of *B. ermanii* stems (**a**) soluble sugar, (**c**) starch and (**e**) NSC as well as *P. jezoensis* stems (**b**) soluble sugar, (**d**) starch and (**f**) NSC (mean ± SD; % of dry matter) along with elevational gradients in Changbai Mountain (*n* = 5 for each elevational site). Different letters indicate significant differences (*p* < 0.05) among elevations as determined by Tukey’s HSD test single-factor ANOVAs.

**Figure 5:**
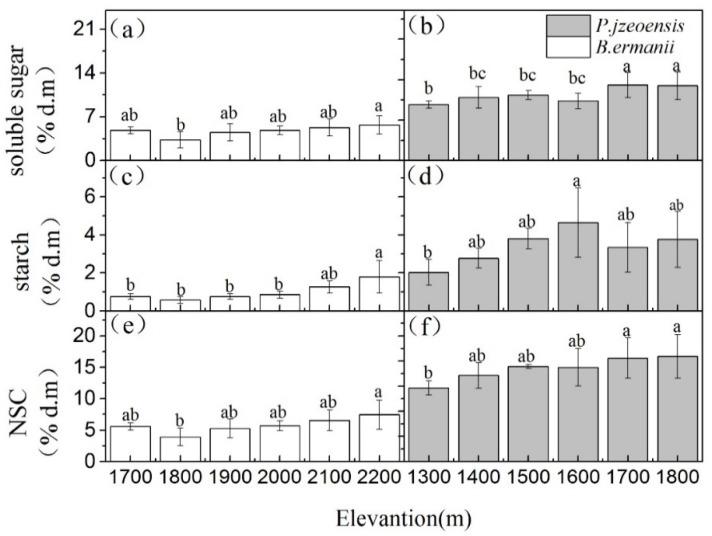
Variation in the concentrations of *B. ermanii* fine roots (**a**) soluble sugar, (**c**) starch and (**e**) NSC as well as *P. jezoensis* stems (**b**) soluble sugar, (**d**) starch and (**f**) NSC (mean ± SD; % of dry matter) along with elevational gradients in Changbai Mountain (*n* = 5 for each elevational site). Different letters indicate significant differences (*p* < 0.05) among elevations as determined by Tukey’s HSD test single-factor ANOVAs.

The concentrations of soluble sugars in *B. ermanii* stems appeared to also remain stable at varying altitudes (Figure 4a), but the starch concentration at high altitudes (2100 m and 2200 m a.s.l.) was significantly higher than that at low altitudes (1700 m and 1800 m a.s.l.) (Figure 4c; *p* < 0.05). Therefore, except for the altitude of 1,800 m, the stems NSC concentrations in the *B. ermanii* did not change with elevation (Figure 4e; *p* > 0.05). Similarly, the elevation did not significantly affect stems NSC, soluble sugars and starch concentrations in *P. jezoensis* (Figure 4b,d,f; *p* > 0.05). Except for maximum value at an altitude of 2200 m a.s.l., no significant differences were observed in the concentrations of soluble sugars and NSC in fine roots of *B. ermanii* with elevation (Figure 5a,e; *p* > 0.05). But fine root starch concentration in *B. ermanii* and NSC, soluble sugars and starch concentrations in *P. jezoensis* tended to increase with increasing elevation (Figure 5b–d,f; *p* < 0.05).

### 2.3. The Trend of Variation in the Soluble Sugars/Starch Ratio with Elevation

**Figure 6:**
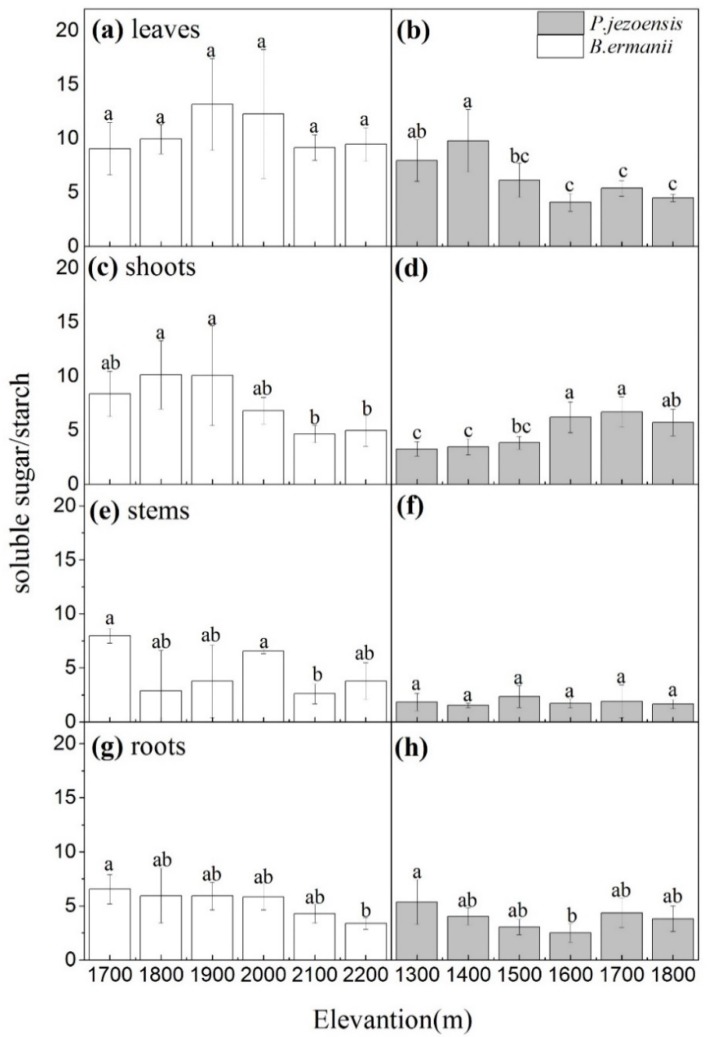
The changes in the soluble sugars/starch ratio (mean ± SD) of *B. ermanii* (**a**) leaves, (**c**) shoots, (**e**) stems and (**g**) roots as well as *P. jezoensis* (**b**) leaves, (**d**) shoots, (**f**) stems and (**h**) roots along with elevational gradients in Changbai Mountain (*n* = 5 for each elevational site and tissue type). Different letters indicate significant differences (*p* < 0.05) among elevations as determined by Tukey’s HSD test single-factor ANOVAs.

The soluble sugars/starch ratio of *B. ermanii* leaves (Figure 6a) and *P. jezoensis* stems (Figure 6f) did not significantly differ with increasing elevation (*p* > 0.05), However, the soluble sugars/starch ratio of the shoots, stems and fine roots in *B. ermanii* were higher at lower elevations (1800 m a.s.l.) than at higher elevations (2100 m and 2200 m a.s.l.) (Figure 6c,e,g; *p* < 0.05). More specifically, a higher value of soluble sugars/starch ratio of *P. jezoensis* leaves and fine roots was observed at the lower elevations (1300 and 1400 m a.s.l.), but the soluble sugars/starch ratio of the shoots tended to increase with increasing elevation (Figure 6b,h,d; *p* < 0.05). Overall, elevation significantly affected the soluble sugars/starch ratio among the tissues of trees, and the different tree species and tissues varied in their response to elevation.

## 3. Discussion

### 3.1. Differences in the NSC Concentrations Between B. ermanii and P. jezoensis

The concentrations of soluble sugars and NSC in the leaves and shoots of the alpine treeline species *B. ermanii* were significantly higher than those of *P. jezoensis* (Figure 1; *p* < 0.05). This is because of different adaptation strategies to high elevations. Compared to evergreen trees, deciduous hardwood leaves are more vulnerable to environmental damage, so the leaves and shoots store higher levels of NSC to enhance their resilience and new leaf growth in the second year [26]. In addition, NSC concentrations is an important indicator that reflects the response of the trees to environmental changes [13,19]. Li et al. (2015) concluded that the concentrations of NSC, soluble sugars and starch of leaves in different forest trees were negatively correlated with temperature [28]. By comparing different tree species in four climatic zones around the world, Korner et al. (2003) found that the concentrations of NSC in the leaves and shoots of trees increased from the tropics to the temperate zone [5]. These results suggest that tree species at a higher elevation with low temperatures tend to have higher NSC concentrations [18,29].

Most studies have shown that the growth and survival of trees at high elevation are not only dependent on NSC concentrations but also require higher soluble sugars/starch ratio (soluble sugar/starch > 3) [19,30]. Higher sugar concentrations are physiological response for trees to survive in the high altitude environments and the soluble sugars/starch ratio can reflect the composition of soluble sugars and starch concentrations as well as mirror the NSC supply level of the trees under given environmental conditions [5]. In our study, the ratio of soluble sugars/starch in the tissues of *B. ermanii* was significantly higher than that of *P. jezoensis* and the soluble sugars/starch ratio in each tissue was > 3, while the soluble sugars/starch ratio in stems of *P. jezoensis* was < 3 (Figure 1d; *p* < 0.05). This is also one of the physiological mechanisms that *B. ermanii* utilizes to adapt to the alpine environment and become a treeline species.

### 3.2. Effects of Elevation on the NSC Storage Strategies of B. ermanii and P. jezoensis

Leaves NSC, soluble sugars and starch concentrations in the *B. ermanii* tended to decrease with increasing elevation (Figure 2a,c,e), while those in *P. jezoensis* remained relatively stable (Figure 2). Two possible causes are: (1) broad-leaved tree species are more susceptible to the stress of high altitude than conifer species, which results in leaf damage and reduces photosynthetic capacity [31] and (2) *B. ermanii* leaves are shed naturally at the end of the growing season and transfer part of the NSC to perennial tissues [27]. While the leaves of *P. jezoensis* usually live for more than 2 years. One-year-old needles store more NSC to resist the stress of high altitude environments and overwintering [26]. Shi et al. (2010) found that the NSC concentrations in the needles of deciduous tree species did not change significantly with elevation, whereas the concentration of NSC in the leaves of evergreen tree species increased significantly [20]. These results suggest that different tree species have different response strategies to environmental changes [20,24,26].

The shoots and stems of *B. ermanii* stored more starch with increase elevation than *P. jezoensis*, which may help to increase its chances of growth and survival at high elevation [22]. In addition, although the concentration of NSC in the stems is low, its biomass is much larger than that of the leaves, shoots and roots, resulting in most NSC in trees storage in stems [26]. In the present study, we found NSC, soluble sugars and starch concentrations in the *P. jezoensis* stems remained stable with elevation, but the starch concentration in the *B. ermanii* stems increased significantly (Figure 4; *p* < 0.05). Therefore, by combining the changes of soluble sugars and starch concentrations in the shoots and stems with elevation, we can predict that the probability of carbon starvation in *B. ermanii* is lower than that in *P. jezoensis* under stress conditions.

Li et al. (2018) studied 11 treeline species around the world and found that the roots of high-altitude trees increased their starch reserves in the summer at the expense of growth, which was an active carbon storage process. In turn, when the photosynthetic products exceeded the demand, it was a passive storage process of NSC [22]. Similarly, we found that the elevation did not significantly effect on the concentration of soluble sugars in the stems and fine roots of *B. ermanii*, but the starch concentration increased significantly with elevation (Figure 5; *p* < 0.05). This indicates that, although the stems and fine roots of *B. ermanii* at high elevation increase NSC storage, they do not have a surplus of soluble sugars, but actively convert the excess soluble sugar into starch. However, the concentrations of soluble sugars and starch in the fine roots of *P. jezoensis* increased significantly with elevation, indicating that *P. jezoensis* provided enough soluble sugars for the growth of fine roots at high elevation. Thus, the increase in starch concentration in the fine roots of *P. jezoensis* may be a passive storage process. Furthermore, trees have self-regulatory mechanisms. When the NSC concentrations increase because of the decrease of the carbon utilization capacity, the trees will reduce the photosynthetic rate through self-regulatory to promote excess carbon utilization [32]. However, Fan et al. (2012) found that photosynthesis in the high-altitude of *B. ermanii* did not decrease [33]. This provides additional evidence that *B. ermanii* increases NSC storage near alpine treeline [34] due to active carbon storage to resist environmental disturbance. This reflects the strategy of adaptation of *B. ermanii* to the high-altitude environment and is one of the reasons it can be distributed in higher altitudes. However, whether the alpine treeline formation is due to reduced carbon utilization resulting in growth restriction must be revisited.

We found that the soluble sugars/starch ratio in different tissues of various tree species displays different trends with elevation and has different reasons. For example, the decrease in the soluble sugars/starch ratio of the roots of *B. ermanii* was due to the significant increase in starch concentration with increasing elevation (Figure 6g), whereas the soluble sugars/starch of *P. jezoensis* shoots increased with altitude was because the proportion of soluble sugar concentration decreased more than that of the starch concentration (Figure 6d). This indicates that under the high altitude environment, whether the soluble sugars/starch ratio can represent the distribution of the minimum threshold needs to be reexamined. Future studies should combine changes in the NSC, soluble sugars, starch concentrations and soluble sugars/starch ratio in different tissues with elevations to reveal the carbon balance, not just only at the organism level.

In addition, *B. ermanii* is one of the elfin tree species on Changbai Mountain [35]. Compared with *P. jezoensis*, *B. ermanii* not only change its shape but actively store starch to resist environmental stress [27]. Therefore, we speculate that *B. ermanii* will move up faster, occupy more space for growth, and the area of the Changbai tundra will decrease with climate warming [3]. Future studies may be necessary to have a systematic discussion on the role of different tree species in the treeline formation. However, because of the complex environment at high altitude, whether our results about the spatial distribution of treeline species are also applicable to other ecosystems and whether the adaptability of other treeline species to the harsh environment is also related to the storage and utilization of NSC need additional study. Overall, our findings emphasize the importance of the carbon storage strategy of tree species for alpine adaptation and a spatial distribution pattern formation.

## 4. Materials and Methods

### 4.1. Study Area

The research area is located in the western slope of the Changbai Mountain Nature Reserve (41°41″49′–42°25″18′ N, 127°42″55′–128°16″48′ E) in Jilin Province of northeastern China. This region has a temperate continental monsoon climate. With the elevation gradient rising from 713 m to 2500 m, the annual mean temperature is from 4.9 °C to −4.8 °C, and the annual precipitation is between 800 mm to 1340 mm, with more than 60% of the precipitation concentrated in the growing season (June–September) [30]. The soil is primarily dark brown forest soil. Under the influence of a mountain climate and terrain, Changbai Mountains formed four distinct zones that range from low elevations to high elevations. The alpine tundra is above 2000 m, the *B. ermanii* forest zone is between 2000 m and 1700 m, the coniferous forest zone is from 1700 m to 1100 m, and the mixed deciduous broad-leaved/conifer forest zone is below 1100 m. *B. ermanii* is primarily distributed at 1700–2200 m, and *P. jezoensis* is primarily distributed at 1100–1800 m [36]. Above the treeline of the *B. ermanii* forest (2000 m), due to the harsh environment, the trees are sparse and dwarf forest [30].

### 4.2. Sampling

At the end of July 2019 (the growing season), six elevation gradients from high to low were selected for sampling based on the distribution of *B. ermanii* and *P. jezoensis* (Table 2). A total of 5 healthy sample trees of similar ages, height and diameters at breast height (DBH) or their basal diameter (at 2100 m and 2200 m) were selected for each elevation, and one-year-old leaves, shoots, stems and fine roots were collected, respectively. The altitude gradient of *B. ermanii* is divided into two parts by the alpine treeline. The trees below 2000 m are more than 2 m high, while those above 2000 m grow to less than 2 m above the alpine treeline. The stem of *B. ermanii* (2100 m and 2200 m) was cut to extract the xylem (1–2 cm). Below the alpine treeline, stem samples of *P. jezoensis* and *B. ermanii* were drilled with a growth cone with a diameter of 5 mm at a height of 1.5 m, and the xylem part 3 cm outside the core was intercepted. To avoid canopy differences, the upper, middle and lower canopy leaves, as well as shoots, were selected to represent the whole canopy. The fine roots (<0.5 cm in diameter with bark) were collected from each of the trees. To reduce the influence of daily temperature range and illumination, all samples were collected at noon and quickly transferred to 4 °C for storage [37]. After the samples were brought back to the laboratory, they were dried for 30 min in an oven at 105 °C and then dried to a constant weight at 65 °C. The dried samples were ground and screened through a 1 mm screen to analyze the NSC.

The growth season average temperatures (100 cm above ground) were measured with a data logger (HOBO, Onset Computer Corporation, Pocasset, Massachusetts, USA) at 1700 m, 2000 m, 2100 m, and 2200 m. Other elevation growth season average temperatures were provided by [38] (Table 2).

### 4.3. NSC Analysis

Soluble sugar and starch concentrations were determined using the anthrone colorimetic method [22]. A total of 0.1 g dried sample was placed in a 10 mL centrifuge tube to which 5 mL 80% ethanol solution was added. The mixture was heated in an 80 °C shaking water bath for 30 min, centrifuged at 4000 rpm for 10 min, and the supernatant was collected in a 25 mL volumetric flask. The precipitate was then extracted twice with 80% ethanol, and the supernatant that had been extracted three times was mixed and added to 25 mL with distilled water to determine the soluble sugar concentration.

The extracted precipitate was placed in an 80 °C shaking water bath to dry the residual alcohol, and 2 mL distilled water was added. The solution was gelatinized in the boiling shaking water bath for 15 min. After being cooled to ambient temperature, 2 mL 9.2 mol/L HClO4 was added, stirred, and the starch was hydrolyzed for 15 min. A volume of 4 mL distilled water was added and centrifuged at 4000 rpm for 10 min to collect the supernatant. A volume of 2 mL 4.6 mol /L HClO4 was added to the precipitate for extraction for 15 min, and 6 mL distilled water was added. After mixing, the supernatant was centrifuged at 4000 rpm for 10 min, and all of the supernatant was collected from each centrifugation. The supernatant was mixed and brought to a volume of 50 mL in a volumetric flask to determine the starch concentration [39]. The soluble sugar and starch were measured at 620 nm in a 721 spectrophotometer (ultraviolet-visible spectrophotometer, Cany Precision Instruments Co., Ltd, Shanghai, China). Soluble sugar and starch concentrations were expressed as the percentage dry weight (% DM).

### 4.4. Data Analysis

Before the data were analyzed, the NSC, soluble sugar and starch data were tested for normality. Two-way ANOVAs were performed with tissue and elevation species as factors to identify the significant effects of NSC, soluble sugars, starch and the soluble sugar/starch ratio. A Tukey HSD test was used to analyze the significance difference of NSC, soluble sugars and starch concentration in different tissues of *B. ermanii* and *P. jezoensis* at different elevations, and the significance level was set as α = 0.05. All the data were analyzed using SPSS 20.0 software (SPSS, Inc., Armonk, NY, USA) and drawn using Origin Pro 8.5 (OriginLab, Northampton, MA, USA).

## Figures and Tables

**Table 1:** Effects of tissue and species on the concentrations of non-structural carbohydrates (NSC), soluble sugar, starch and soluble sugars/starch, tested using a mixed effects model. The treeline was used as a random variable to account for between-treeline variance. The significant levels of *p* < 0.05 are shown in bold.

	Soluble Sugar		Starch		NSC		Soluble Sugar/Starch	
	F	P	F	P	F	P	F	P
Species (S)	7.70	0.006	105.40	<0.000	32.79	<0.000	91.93	<0.000
Tissue (T)	389.90	<0.000	60.26	<0.000	306.37	<0.000	59.87	<0.000
T × S	122.18	<0.000	32.61	<0.000	110.64	<0.000	3.76	0.011

**Table 2:** Characteristics of the plots and the sampling trees *Betula ermanii* (mean ± standard deviation; *n* = 5 trees), *Picea jezoensis* (mean ± standard deviation; *n* = 5 trees) located in the Changbai Mountain.

Species	Elevation	Growth Season Average Temperature (°C)	Average		Slope Exposure
	(m)		DBH (cm)	Hight (m)	
*Betula ermanii*	2200	9.88	1.1 ± 0.1 ^a^	1.0 ± 0.1	West
	2100	10.68	1.1 ± 0.1 ^a^	1.5 ± 0.1	West
	2000	11.50	4.8 ± 0.4	4.1 ± 0.1	West
	1900	12.04	9.4 ± 1.0	9.3 ± 0.4	West
	1800	12.58	9.2 ± 0.7	9.2 ± 0.5	West
	1700	13.12	10.9 ± 0.7	11.2 ± 0.9	West
*Picea jezoensis*	1800	12.58	24.5 ± 1.2	10.8 ± 0.5	West
	1700	13.12	24.7 ± 1.3	10.9 ± 0.5	West
	1600	13.57	24.6 ± 1.2	11.0 ± 0.5	West
	1500	13.94	24.5 ± 1.4	10.5 ± 0.7	West
	1400	14.31	24.2 ± 0.9	10.7 ± 0.6	West
	1300	14.68	23.8 ± 0.9	11.1 ± 0.9	West

^a^: Basal diameter (about 1 cm above the ground surface).
